# Laparoscopic Removal of Retained Surgical Sponge After Caesarean Section: A Case Report

**DOI:** 10.7759/cureus.21375

**Published:** 2022-01-18

**Authors:** Archana Khanduri, Jyoti Gupta, Houssem Ammar, Rahul Gupta

**Affiliations:** 1 Gastrointestinal Surgery, Synergy Institute of Medical Sciences, Dehradun, IND; 2 Radiation Oncology, Himalayan Institute of Medical Sciences, Dehradun, IND; 3 Surgery, Sahloul Hospital, Sousse, TUN

**Keywords:** foreign body, textiloma, caesarean section, laparoscopy, gossypiboma

## Abstract

Gossypiboma or textiloma denotes the formation of a mass lesion due to the development of foreign body reactions around the retained surgical item in the body. Most of the cases are asymptomatic in the initial postoperative period. Due to the dense adhesions, most of the cases of intra-abdominal gossypiboma are treated by an open approach. Here we present a case of a 38-year-old lady with left iliac fossa pain one month after caesarean section. Contrast-enhanced computed tomography of the abdomen revealed gossypiboma. The patient was successfully treated with laparoscopic removal of the gossypiboma.

## Introduction

The term gossypiboma is used to describe surgical sponges or gauze retained inside a patient’s body after a surgical procedure [1-4]. The body reacts to the retained foreign body by an exudative inflammatory or aseptic fibrotic reaction to encapsulate the cotton material, which results in the formation of an intra-abdominal mass [1,4].

This unintentional but avoidable surgical complication causes serious damage to the patient’s health. The current estimate of retained surgical items is one in 10,000, with surgical sponges accounting for the majority of cases. However, the exact incidence is not known as it is under-reported due to the fear of medicolegal consequences. The most common sites of gossypiboma are the abdomen (56%), pelvis (18%), and thorax (11%) [5].

The foreign body should be removed once diagnosed as it can erode in the adjoining structures leading to organ damage and fistula formation [6-8]. Here, we report a case of post-caesarean section gossypiboma treated successfully by laparoscopy.

## Case presentation

**Figure 1:**
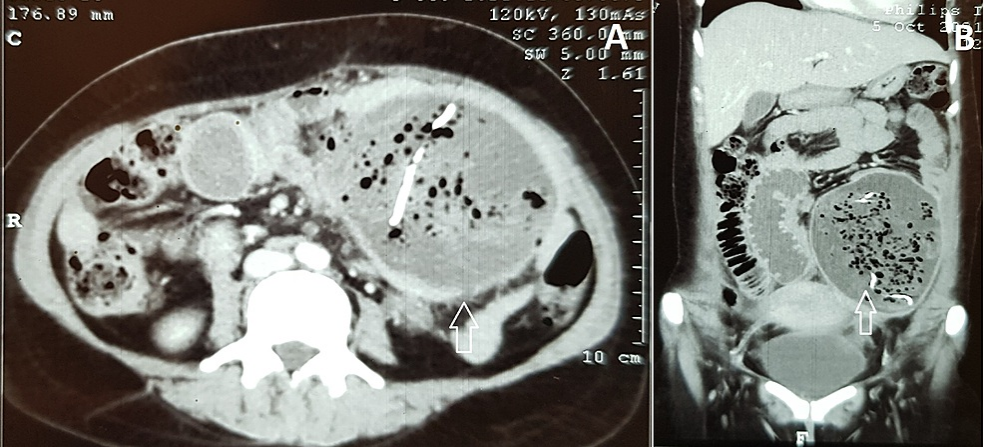
Contrast enhanced computed tomography of the abdomen and pelvis in axial (A) and coronal (B) section showing the large heterogenous lesion in the left iliac fossa containing air foci and linear radio-opaque structures (arrows) suggestive of gossypiboma.

A 38-year-old female presented with left lower quadrant pain for seven days associated with low-grade fever. There was no history of vomiting, altered bowel habits, hematemesis, melena, or weight loss. She had undergone a lower segment caesarean section one month back. On clinical examination, she was febrile and had mild tenderness in the left lower quadrant of the abdomen. The previous Pfannenstiel incision of the caesarean section had completely healed with no palpable lump or cough impulse. Routine blood investigations were within normal limits. Abdominal ultrasonography revealed a bulky uterus with a 42 cc collection in the anterior abdominal wall at the left lateral end of the Pfannenstiel scar. There was an irregular-shaped large echogenic mass of 109 x 77 mm with posterior acoustic shadow in the left iliac fossa raising the suspicion of gossypiboma. In contrast-enhanced computed tomography (CECT) of the abdomen, a large well-defined peripherally enhancing hypodense lesion measuring 12 x 11 x 9 cm with multiple small air foci were noted in the left iliac fossa causing mass effect and compression of the adjoining small and large bowel loops suggestive of gossypiboma (Figure 1). In view of the above findings, the patient was planned for diagnostic laparoscopy and removal of gossypiboma. 

**Figure 2:**
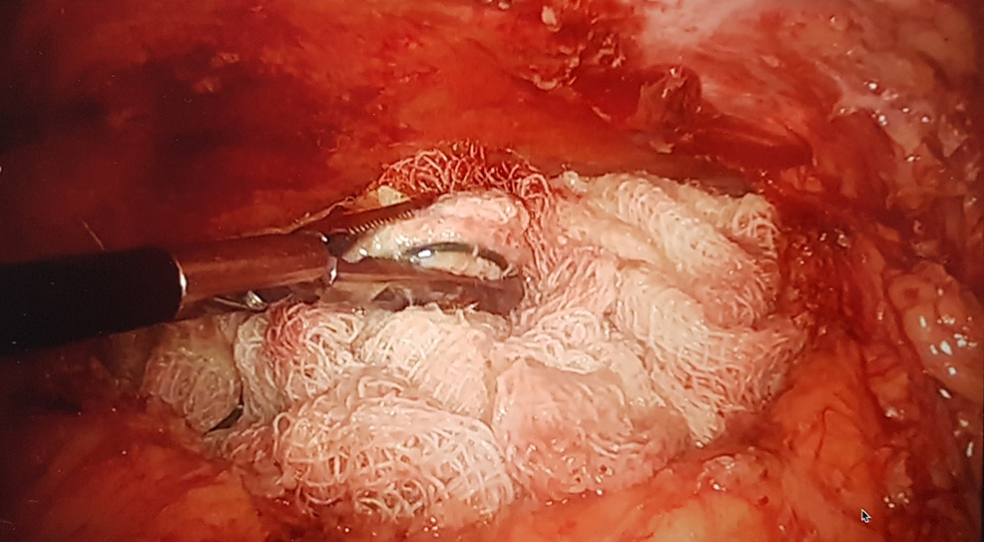
Intraoperative photograph showing the gossypiboma.

Pneumoperitoneum was created by Veress needle through Palmer’s point. The 10 mm camera port was placed in the right upper quadrant, and two 5 mm working ports were placed in the right iliac fossa and epigastrium, respectively. On diagnostic laparoscopy, the small and large bowel loops densely adhered to the anterior abdominal wall in the left iliac fossa. Adhesiolysis was done, and the cavity was opened. About 100 ml of seropurulent fluid was drained. A retained surgical sponge was seen densely adhered to the cavity lined by granulation tissue (Figure 2).

With sharp and blunt dissection, the surgical sponge was separated from the wall of the cavity. Thorough lavage was given. No leakage of bile or enteric content from the wall of the cavity was noted. A tube drain was placed in the cavity, and the retained surgical sponge was extracted through a small lower midline incision. The operative time was 120 minutes with a blood loss of 50 ml. The postoperative course was uneventful, with a hospital stay of five days. The drain was removed on the fifth postoperative day before discharge as the output was minimal. Pus culture was sterile. On the last follow up at three months after surgery, the patient was doing well without any complications.

## Discussion

Gossypiboma is derived from the words “Gossypium” and “boma”, which means ‘cotton wool’ and ‘to coverup’, respectively. The first case was described by Wilson et al. in 1884. The incidence of gossypiboma varies from one in 8801 to one in 18760 of inpatient operations [9]. However, the actual incidence is not known as it is underreported due to the possible legal ramifications of this technical oversight [10]. The most frequent sites are abdominal (74%) and thoracic (11%) cavities, respectively [1,2,5]. A surgical sponge is the most frequently retained surgical foreign body (69%), while other retained items include forceps, electrodes, needles, and retractors [5,9,11]. Gossypiboma has been reported after all types of surgeries, including gastrointestinal [2,12], gynecological [1,3,6], urological, vascular, orthopaedic, and spinal surgeries [13]. Gawande et al. identified emergency procedures, unplanned changes in the surgical procedure, and obesity as the risk factors for the occurrence of retained sponges [14]. 

Most of the patients are asymptomatic in the early postoperative period. However, the retained surgical items can erode the surrounding structures and lead to fistula formation with the gastrointestinal and genitourinary tract [1]. Once the gossypiboma starts eroding, the patients often develop symptoms leading to its detection. Rarely, they remain asymptomatic for many years and lead to life-threatening complications such as bleeding, perforation, and obstruction [6-8].

In the immediate postoperative period, a plain radiograph may show the radio-opaque line present in the sponges [2]. However, the radio-opaque may not be visible in up to 10% of cases. Further investigations such as ultrasound, CECT, and magnetic resonance imaging (MRI) are required to confirm the diagnosis. On ultrasound, they appear as an echogenic mass with acoustic shadow [2]. On CECT, gossypiboma can be seen as a heterogenous mass containing radio-opaque structures surrounded by enhancing capsules [6]. In some cases, the air foci can be seen within the lesion on CT, as seen in the present case [6]. On MRI, the lesions have low to intermediate signal intensity on T1-weighted images and high signal intensity on T2-weighted images [13]. However, the diagnosis is confirmed only at the surgery. 

During surgical exploration, the gossypiboma is usually covered by the surrounding structures, with dense adhesions as seen in the present case. In long-standing cases, there may be partial or complete migration of the retained surgical sponge into the adjoining hollow viscus [6-8]. The foreign-body reaction makes the surgical removal of gossypiboma difficult, especially by minimally invasive technique. There are very limited reports on laparoscopic removal of the gossypiboma [1-3]. However, if feasible, laparoscopy offers the advantages of decreased postoperative pain, wound-related complications, and faster recovery. 

A retained sponge is associated with significant morbidities. Hence, a systematic approach during the operation is essential to reduce its incidence. The first step is to perform sponge counts multiple times during surgery. The sponge count must be performed prior to the start of any procedure, at the time of addition of a new sponge, prior to the closure of any cavity, and at the time of closure of the incision. If any discrepancy is found, the surgical team should look for the missing item [15]. The American College of Surgeons endorses the same view and emphasizes the use of a surgical safety checklist as a part of the institutional policy to improve surgical safety and patient care [16]. 

## Conclusions

Gossypiboma is an avoidable surgical complication. Intraoperative gauze counting and double-checking with the surgical team is crucial to facilitate early recognition and detection of any missed sponge or instrument to decrease its occurrence. Gossypiboma can have serious consequences on the patients’ health and lead to medicolegal challenges for the treating surgeons. Once detected, it should be surgically removed. Laparoscopy can be an excellent approach for the retrieval of gossypiboma in appropriately selected patients.
